# Bowel ischemia as onset of COVID‐19 in otherwise asymptomatic patients with persistently negative swab

**DOI:** 10.1111/joim.13385

**Published:** 2021-10-08

**Authors:** Paolo Zamboni, Daria Bortolotti, Savino Occhionorelli, Luca Traina, Luca Maria Neri, Roberta Rizzo, Roberta Gafà, Angelina Passaro

**Affiliations:** ^1^ Department of Translational Medicine University of Ferrara Ferrara Italy; ^2^ Department of Surgery University Hospital of Ferrara Arcispedale Sant'Anna Ferrara Italy; ^3^ Department of Chemical and Pharmaceutical Sciences University of Ferrara Ferrara Italy; ^4^ Oncological and Medical Department University Hospital of Ferrara Arcispedale Sant'Anna Ferrara Italy; ^5^ Medical Department University Hospital of Ferrara Arcispedale Sant'Anna Ferrara Italy

**Keywords:** bowel ischemia, COVID‐19, SARS‐CoV‐2, vascular event

## Abstract

**Background:**

Asymptomatic patients with Severe Acute Respiratory Syndrome Coronavirus 2 (SARS‐CoV‐2) can develop hypercoagulable conditions and acute vascular events. The objective of this study is to determine whether SARS‐CoV‐2 was present in resected specimens from patients with acute bowel ischemia, but asymptomatic for Coronavirus Disease 2019 (COVID‐19) and with persistently real‐time polymerase chain reaction negative pharyngeal swab.

**Methods:**

Three consecutive patients presented severe abdominal symptoms due to extensive ischemia and necrosis of the bowel, with co‐existent thrombosis of abdominal blood vessels. None had the usual manifestations of COVID‐19, and repeated pharyngeal swabs tested negative. They underwent emergency surgery with intestinal resection. Immunohistochemical testing for SARS‐CoV‐2 on resected tissue was performed.

**Results:**

All tested samples were strongly positive for SARS‐CoV‐2.

**Conclusions:**

This is the first case report in which patients with severe intestinal symptoms presented a marked SARS‐CoV‐2 positivity in the resected tissues, without any usual clinical manifestations of COVID‐19. These results suggest that the patients might be infected with SARS‐CoV‐2 presenting acute abdominal distress but without respiratory or constitutional symptoms.

## Graphical abstract

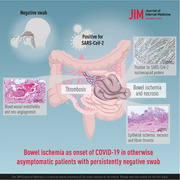

**Abbreviations**
COVID‐19: Coronavirus Disease 2019
CT: computed tomography
IgA: Immunoglobulin A
IHC: immunohistochemistry
RNA: ribonucleic acid
RT‐PCR: real‐time polymerase chain reaction
SARS‐CoV‐2: Severe Acute Respiratory Syndrome Coronavirus 2

## Background

It is estimated that around 80% of people infected with the Severe Acute Respiratory Syndrome Coronavirus 2 (SARS‐CoV‐2) virus have no symptoms or very mild symptoms [1]. Since the literature data on CoronaVirus Disease 2019 (COVID‐19) are mainly based on the evaluation of hospitalized patients, including those requiring intensive care [1, 2, 3], few data are available on the clinical course of COVID‐19 in asymptomatic cases.

It is now evident that SARS‐CoV‐2 infection affects endothelial cells, which are rich in angiotensin‐converting enzyme 2 receptors used by the virus to enter the cells. The endothelium of large or small, arterial or venous, blood vessels seems to be a target for SARS‐CoV‐2 infection [4, 5, 6]. Furthermore, SARS‐CoV‐2 infection often induces an unexplained hypercoagulable state, leading to severe and potentially fatal vascular events, as venous thromboembolism [4, 5, 6, 7, 8, 9, 10, 11, 12, 13] of cerebral and lower limb arteries [14, 15].

Literature data support the implication of SARS‐CoV‐2 infection in the manifestation of major adverse cardiovascular events, symptomatic venous thromboembolism and major arterial or venous thromboembolism in patients with COVID‐19, especially in the intensive care setting, despite the use of thromboprophylaxis [16, 17]. On the contrary, no data are available on patients with acute abdominal symptoms and oro/nasopharyngeal samples test negative for SARS‐CoV‐2.

In this report, we analyzed resected specimens from three consecutive patients with acute abdominal symptoms and no signs of interstitial pneumonia and oro/nasopharyngeal swabs persistently negative for SARS‐CoV‐2. We performed SARS‐CoV‐2 immunohistochemistry (IHC) on resected material, with the aim to clarify the possible SARS‐CoV‐2 infection associated with these clinical cases.

## Materials and methods

### Patients

**Fig. 1:**
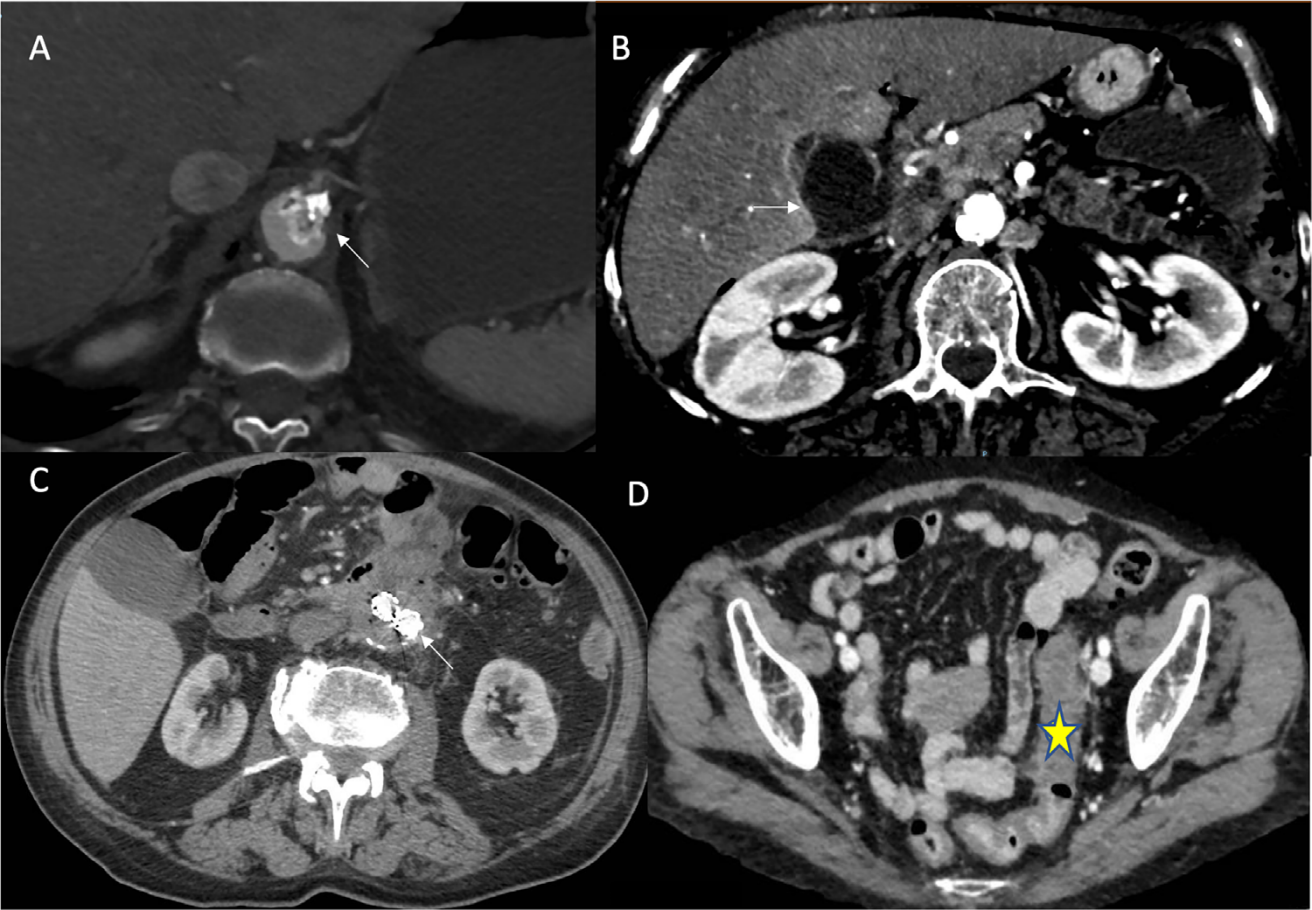
(a) Case 1: Aortic thrombosis at the origin of celiac trunk (arrow). (b) Case 1: Gangrenous gallbladder (arrow). (c) Case 2: Duodenum and abdominal aorta fused at the level of aneurism. Note fluid and air accumulation within aneurism cavity and aorto‐bi‐iliac endograft (arrow). (d) Case 3: Edema of the sigmoid colon (star) with perivisceral fluid accumulation

Patient 1 was a 76‐year‐old hypercholesterolemic female heavy smoker who had received a mastectomy for breast cancer 30 years previously. On admission, she had abdominal pain, vomiting and rebound tenderness. White blood cells were 15.76 × 10^3^/μl; lactate dehydrogenase, 293 U/L; negative swab test for SARS‐CoV‐2 and mild pleural effusion but no interstitial pneumonia on chest computed tomography (CT). Abdominal CT showed that the ileum in the right iliac fossa was distended, with fluid accumulation in the abdomen. Thromboses were present on an atheromatic plaque within the celiac trunk and the aorta below the kidneys (Fig. 1). The superior and inferior mesenteric arteries were obstructed close to their origins so that intestinal blood supply was entirely from the celiac trunk. Around 60 cm of necrotic ileum was removed on laparotomy. Fever and abdominal pain (right hypochondrium) developed on the third postoperative day. Abdominal CT showed abdominal fluid accumulation and distended, radiologically heterogeneous, gallbladder (no gallstones), surrounded by fluid (Fig. 1 and Fig. S1). A laparotomic approach was used to remove the gallbladder (which proved gangrenous, probably caused by coeliac trunk embolism) and wash and drain the peritoneum. The extensive thromboses involving abdominal arteries led us to suspect SARS‐CoV‐2 and hence investigate viral presence on resected specimens. Over the subsequent 35 days, five swabs were taken, all but one was negative for SARS‐CoV‐2. The patient was discharged 45 days after admission with a subclavian catheter for parenteral nutrition because of abdominal angina. She died at home 4 months later.

Patient 2 was a 79‐year‐old male with ischemic cardiopathy and dilated ventricular apex treated with oral dicumarol. An abdominal aorta aneurism had been repaired with an aorto‐bi‐iliac endograft. On admission he had abdominal pain and elevated temperature (38.3°C); hemoglobin, 8.6 g/dl; white cells, 1.40 × 10^3^/μl; procalcitonin, 79 ng/ml; C‐reactive protein, 25 mg/dl; prothrombin time, 4.16 s and D‐dimer, 3.38 μg/ml. Thoracic CT was unremarkable and he tested negative for SARS‐CoV‐2.

Abdominal CT revealed a fluid accumulation containing small air bubbles within the cavity of the aortic aneurism. At this level, the aorta was tightly fused to the duodenal wall (Fig. 1 and Fig. S1). On laparotomy, ischemic necrosis was found to involve the distal fourth of the duodenum, which appeared connected to the aneurism cavity via fistulae. The aortic prosthesis was undamaged and there was no endo‐leakage. The necrotic duodenum and first loop of the jejunum were removed, followed by duodenojejunal anastomosis. Postoperatively the patient developed bacterial and fungal infections and was not discharged until 38 days after admission. While in hospital, weekly tests were always negative for SARS‐CoV‐2. However, it is worthwhile testing the resected samples for a virus as the patient was from Bergamo, the Italian city with the highest number of COVID‐19 deaths. Three months after discharge, CT showed that the aortic graft continued to function well.

Patient 3 was a 64‐year‐old woman admitted for severe abdominal pain. SARS‐CoV‐2 test was negative and chest CT was unremarkable. Hemoglobin, 8.6 g/dl; white cells, 1.20 × 10^3^/μl; D‐dimer, 4.4 μg/ml; C‐reactive protein 32 mg/dl; troponin I, 448 ng/L and B‐type natriuretic peptide, 230 pg/ml. Abdominal CT revealed edematous thickening of the sigmoid colon with an accumulation of fluid and air (Fig. 1 and Supporting Information). The inferior mesenteric artery was not present after its origin. The hypogastric and right femoral veins were thrombosed. Laparotomy revealed acute ischemia and edema of the sigmoid colon, which was also perforated with retroperitoneal fluid accumulation. Laparotomic Hartmann sigmoidectomy was performed. Sepsis developed postoperatively and the patient died of multiple organ failure 11 days after admission. The extensive venous thrombosis in the absence of acquired or hereditary thrombophilia and in the presence of high D‐dimer prompted us to examine the resected tissue for SARS‐CoV‐2.

Ileal, colon and gallbladder control samples were obtained, respectively, from a 78‐year‐old female with intestinal ischemia (surgical resection August 2019), from an 83‐year‐old female with colonic ischemia (surgical resection February 2019) and from a 74‐years‐old male with acute gangrenous cholecystitis (surgical resection March 2019).

### Histology and immunohistochemistry

Tissue samples were fixed in formaldehyde and embedded in paraffin. For histological examination, slides were stained with hematoxylin and eosin. IHC analysis was performed on 4‐μm thick sections: following antibody retrieval (by heating the samples in citrate buffer pH6 with the 0.05% tween20), the slides were treated with anti‐SARS‐CoV‐2 nucleocapsid protein (Novus Biologicals, Centennial, 1:250 dilution) or anti‐isotype control (Novus Biologicals). Positivity was detected by horseradish peroxidase/3,3′‐diaminobenzidine by UltraTek HRP (anti‐polyvalent) Ready‐To‐Use (ScyTech Laboratories) [18]. Ileum and colon samples obtained in the pre‐COVID‐19 era were not positive for SARS‐CoV‐2 (Fig. S2).

Results

**Fig. 2:**
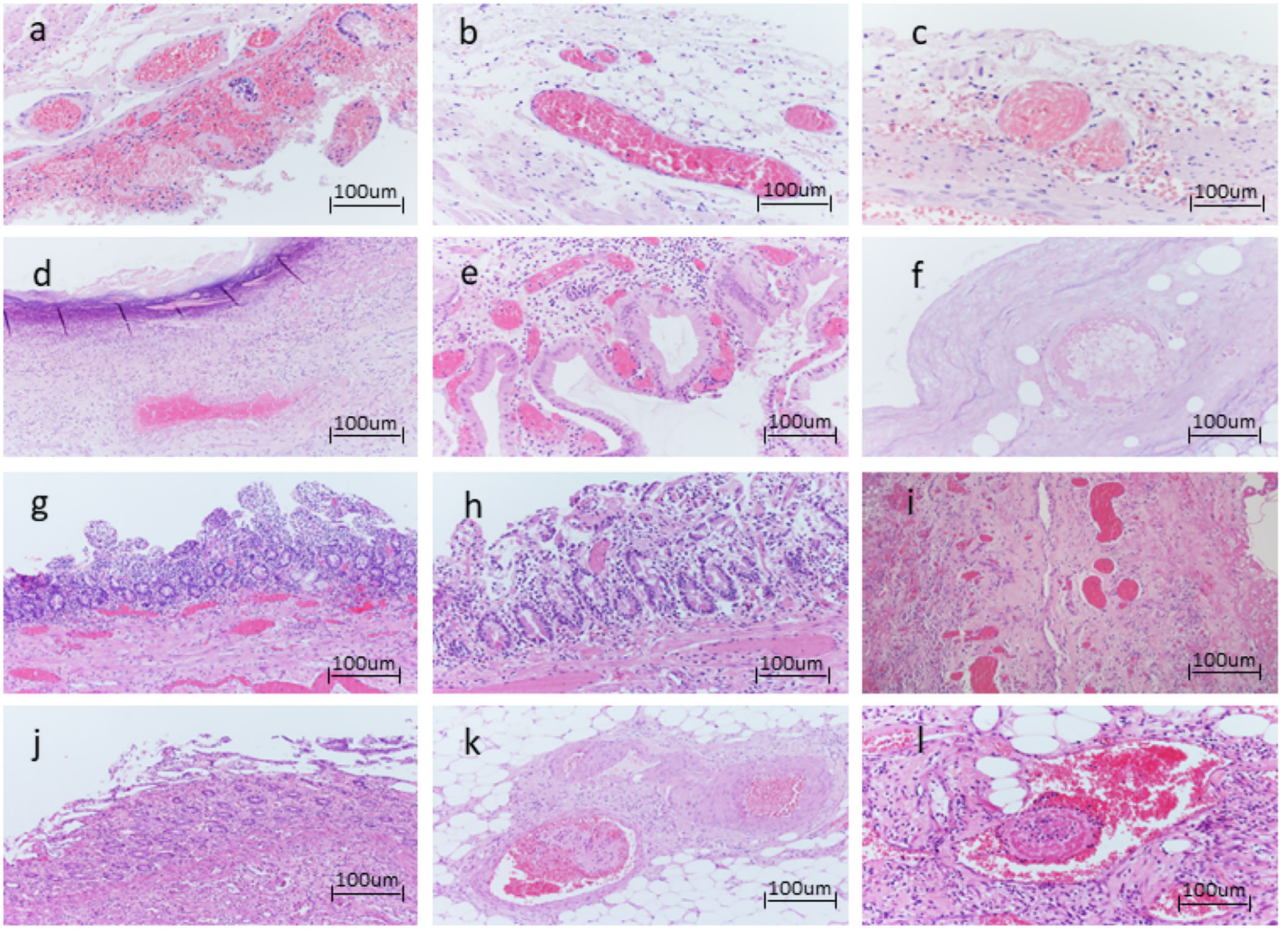
Case 1: Optical microscopy. (a) Hemorrhagic necrosis of ileal mucosa with focal crypts preservation (hematoxylin–eosin (H‐E), 20x). Fibrin thrombi (b) H‐E, 20x and (c) H‐E, 40x. Case 1: Optical microscopy. (d) Acute gangrenous cholecystitis. (e) Gangrenous necrosis (H‐E, 10x) with (f) several mural thrombi (H‐E, 10x). Case 2: Optical microscopy. (g) Ischemic sigma with atrophic crypts and aspects of neo‐angiogenesis (H‐E, 10x), h) fibrin thrombi (H‐E, 10x) and (i) aspects of sustained endothelial inflammation (H‐E, 10x). Case 3: Optical microscopy. (j) Patchy ischemia of the duodenum with surface epithelial necrosis (H‐E, 10x) and (k) H‐E, 20x. (l) Fibrin thrombi (10x).

**Fig. 3:**
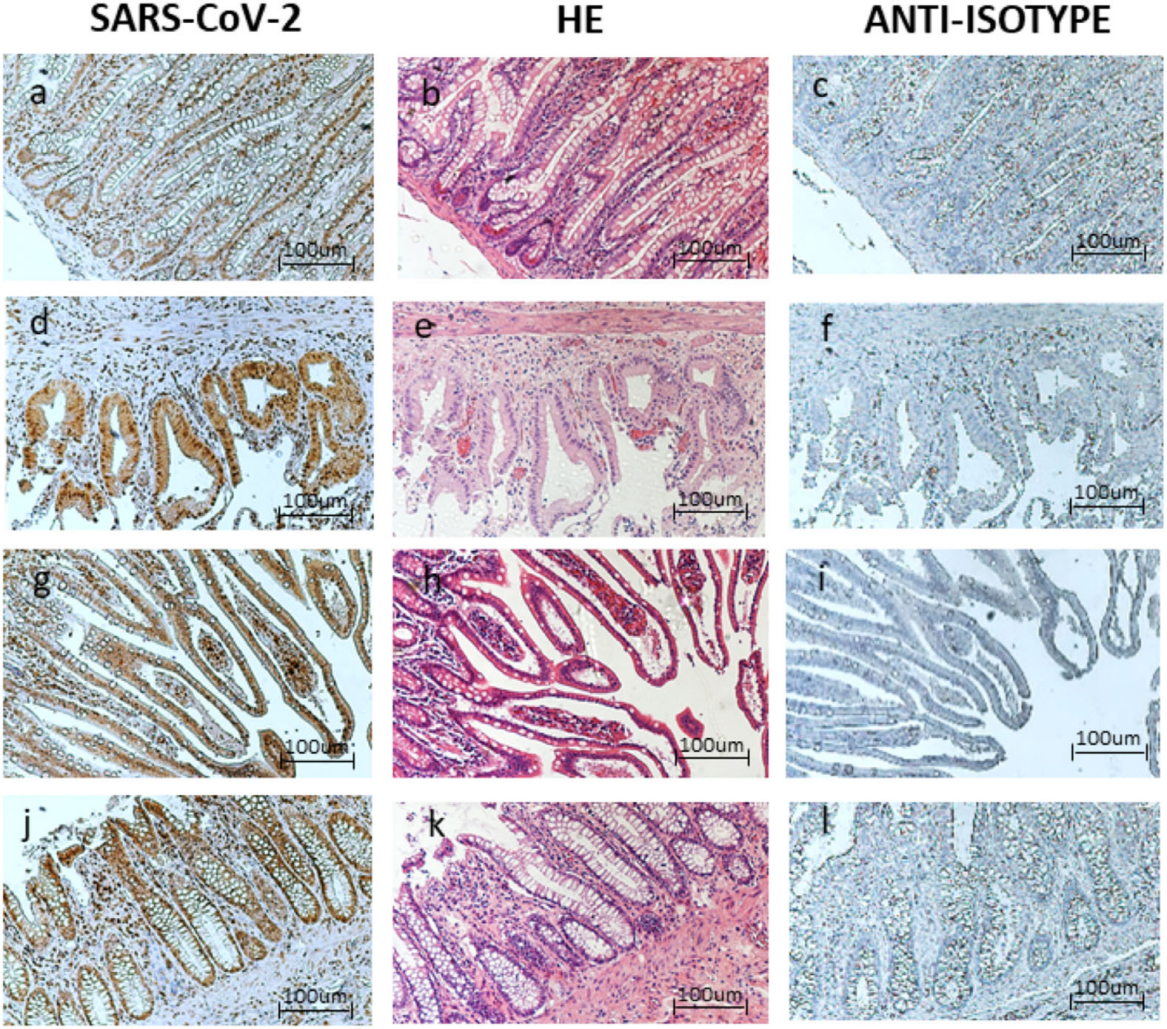
Case 1: Optical microscopy. Immunohistochemistry (IHC), 10x positive staining for (a) Acute Respiratory Syndrome Coronavirus 2 (SARS‐CoV‐2) virus, (b) hematoxylin–eosin, (c) anti‐isotype staining. Case 1: Optical microscopy. IHC, 10x positive staining for (d) SARS‐CoV‐2 virus, (e) hematoxylin‐eosin, (f) anti‐isotype staining. Case 2: Optical microscopy. IHC, 10x positive staining for (g) SARS‐CoV‐2 virus, (h) hematoxylin‐eosin, (i) anti‐isotype staining. Case 3: Optical microscopy. IHC, 10x positive staining for (j) SARS‐CoV‐2 virus, (k) hematoxylin eosin, (l) anti‐isotype staining.

Histological examination of all samples showed extensive intestinal and gallbladder wall ischemia and necrosis, with thrombosis of intramural blood vessels (Fig. 2). In particular, in Fig. 2a, there is hemorrhagic necrosis of ileal mucosa with focal crypt preservation and fibrin thrombi (Fig. 2a–c). In Fig. 2d, there is an evident acute gangrenous cholecystitis with several thrombi (Fig. 2e,f). In Fig. 2g,h, there is a patchy ischemia of the duodenum with surface epithelial necrosis and fibrin thrombi (Fig. 2i). In Fig. 2j, there is an ischemic colitis with atrophic crypts and fibrin thrombi (Fig. 2k,l). Blood vessel endotheliitis and neo‐angiogenesis were also observed, particularly in samples from patient 3 (Fig. 2j–l). Since the ischemic area might present an aspecific staining, we examined the adjacent tissue area for SARS‐CoV‐2 nucleocapsid protein presence. The hematoxylin–eosin staining showed that the area used for the SARS‐CoV‐2 nucleoprotein staining was not characterized by evident necrosis and inflammation, which might cause false‐negative results (Fig. 3b,e,h and k). All the investigated samples presented SARS‐CoV‐2 positive areas. In particular, Fig. 3a shows a cross‐sectional view of ileal villi after IHC staining with anti‐SARS‐CoV‐2 nucleoprotein. The positivity was evident in both the epithelium and in the lamina propria. In Fig. 3d, the cholecystitis showed an epithelial localization of SARS‐CoV‐2 nucleoprotein staining. In Fig. 3g, the sigma epithelium was positive for SARS‐CoV‐2 nucleoprotein as the duodenum in Fig. 3j. The anti‐isotype control confirms the absence of aspecific staining. Similarly, SARS‐CoV‐2 nucleoprotein staining was totally absent in the ileal, colon and cholecystitis section from patients of a pre‐COVID19 period, as reported in the Materials and Methods section (Fig. S2). These data confirm the specificity of the SARS‐CoV‐2 nucleoprotein staining.

## Discussion

The results reported in this research suggest that some patients, characterized by abdomen leading to intestinal infarction and necrosis, even in the absence of the usual (respiratory or otherwise) symptoms and signs of COVID‐19, and negative oro/nasopharyngeal samples, might present SARS‐CoV‐2 infection in the resected tissues. The thrombosis, endothelitis and neo‐angiogenesis that we observed in the small blood vessels of intestinal submucosa (Fig. 2) were closely similar to those described in the lungs of COVID‐19 patients [4], supporting the correlation between SARS‐CoV‐2 infection and gastrointestinal symptoms. To our knowledge, this acute and severe intestinal onset has never been reported, even if intestinal infarction was reported as a complication of interstitial pneumonia in patients testing positive for SARS‐CoV‐2 [19, 20, 21, 22, 23, 24, 25] and in patients with COVID‐19‐like pneumonia, but a SARS‐CoV‐2 negative swab [26].

As a confirmation of the possible involvement of the gastrointestinal tract in COVID‐19 patients, literature data report nausea/vomiting symptoms in 5.0% and diarrhea in 3.8% of COVID‐19 patients [3]. However, SARS‐CoV‐2 presence was not previously reported in the gastrointestinal samples [20, 21, 26].

It seems unlikely that a virus that gains entry to the body via the respiratory tract and whose typical manifestations are mainly respiratory should primarily infect the gut. However, in COVID‐19 patients whose respiratory symptoms have subsided, the virus may persist in the intestines for an extended period. In a meta‐analysis, viral RNA was found in the feces of 48% (95% CI, 38.3–57.9) of COVID‐19 patients, even if the 70.3% (95% CI, 49.6–85.1) was SARS‐CoV‐2 negative in oro/nasopharyngeal samples [27, 28].

We can hypothesize that these three patients were infected with SARS‐CoV‐2, which remained asymptomatic at the respiratory level, but evolved in gastrointestinal pathogenesis. The negative results of the oro/nasopharyngeal samples might be due to the limit of detection of the test, especially in late infections [29]. The presence of the virus in the gastrointestinal tissues suggests the transmission of the infection from the respiratory tract to the gastrointestinal compartment via intestinal blood vessels, perhaps in relation to declining levels of neutralizing antibodies, particularly immunoglobulin A (IgA). IgA is principally expressed in the body's mucous membranes, particularly in the respiratory and gastrointestinal tracts where IgA is involved in the control of viral infections [30] as the earliest neutralizing antibody response to SARS‐CoV‐2 infection [31]. These three cases might be representative of an acute gastrointestinal infection/re‐infection with SARS‐CoV‐2 that developed in severe gastrointestinal symptoms without affecting the respiratory tract.

These data suggest the fecal‐oral infection/re‐infection as a possible alternative way of SARS‐CoV‐2 spreading, which deserves further investigation. In fact, even more, evidence suggests that, like other related viruses, SARS‐CoV‐2 may also be an enteric virus that can infect via the fecal‐oral route, possibly via angiotensin‐converting enzyme 2 expression in the gut [32]. Such a hypothesis, together with the presence of SARS‐CoV‐2 RNA in the feces of COVID‐19 patients, underlines the possible presence of potentially infectious materials [33], such as stools or baby diapers, that might increase the viral persistence in the environment.

## Conclusion

In conclusion, our results underline the importance to evaluate the possible presence of SARS‐CoV‐2 gut infection during the clinical practice, since patients without evident respiratory symptoms might develop severe abdominal events due to gut infection. We are aware of the limit of this research, due to the limited number of samples, that deserve further investigation. However, we hypothesize that the gastrointestinal tract can act as a virus reservoir even when the oro‐nasal swab is negative. We suggest testing for SARS‐CoV‐2 presence in the resected samples of recent emergency cases of intestinal ischemia. This evaluation might increase the number of victims of the SARS‐CoV‐2 pandemic than currently suspected.

## Acknowledgments

The authors thank Don Ward, BSc, for help with the English; Roberto Galeotti, MD, Associate Professor of Radiology, for his lecture on CT imaging and Cristina Bosi, BSc, for preparing material for the histologic and immunohistochemical analyses. The research was supported by COVID‐19 grant from the University of Ferrara and by crowdfunding campaign from University of Ferrara.

Open Access Funding provided by Universita degli Studi di Ferrara within the CRUI‐CARE Agreement. [Correction added on 11 May 2022, after first online publication: Projekt CRUI‐CARE funding statement has been added.]

## Conflict of interest

The authors declare that they have no conflict of interest.

## Supporting information

Figure 1. Case 1. A Left: Chest CT with pleural effusion but negative for interstitial pneumonia. B Middle: Aortic thrombosis of origin of the celiac trunk. C Right: Pervious vessels of the celiac trunk. Case 1. D Left: Re‐canalized superior mesenteric artery. E Middle: Sub‐renal aortic thrombosis. F Right: Gangrenous gallbladder. Case 2. G Left fused duodenum and abdominal aorta at the level of aneurysm, note fluid and air accumulation within aneurism cavity and aorto‐bi‐iliac endoprosthesis. Case 3. H Middle: Edema of sigmoid colon with the perivisceral fluid collection. I Right: Thrombotic right hypogastric vein.

Figure 2. Colon section. Optical microscopy. IHC 10X positive staining for (a) SARS‐CoV‐2 virus, (b) anti‐isotype staining. Cholecystitis section. Optical microscopy. IHC 10X positive staining for d) SARS‐CoV‐2 virus, e) anti‐isotype staining. Ileum section. Optical Microscopy. IHC 10X positive staining for g) SARS‐CoV‐2 virus, h) anti‐isotype staining.
