# Supplementary figures and images for: Bowel ischemia as onset of COVID‐19 in otherwise asymptomatic patients with persistently negative swab
Source: J Intern Med. 2021 Oct 8;291(2):224–31. doi: 10.1111/joim.13385 (PMC8662187; doi:10.1111/joim.13385)

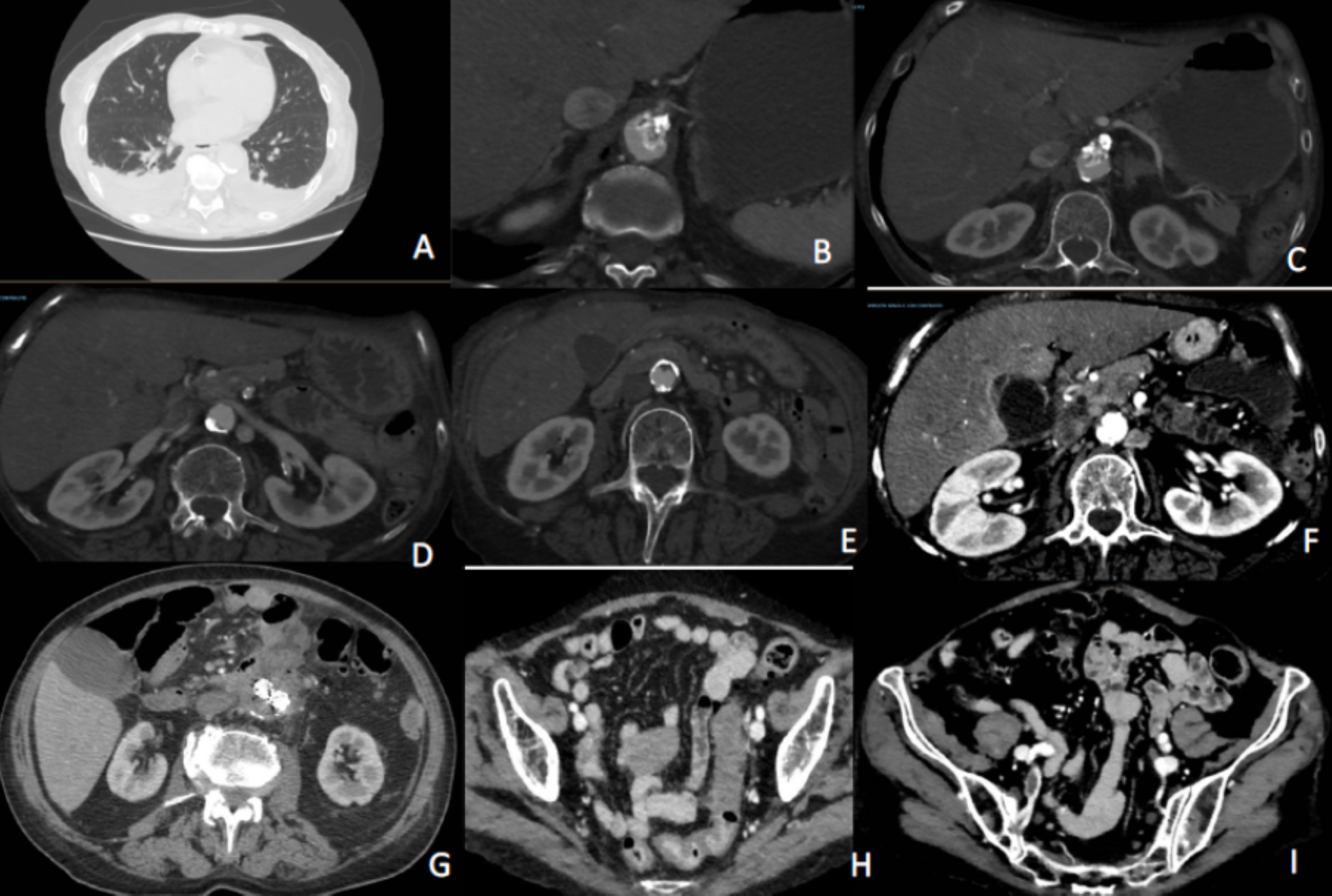

Supplement: Supplementary file 1 — Figure 1. Case 1. A Left: Chest CT with pleural effusion but negative for interstitial pneumonia. B Middle: Aortic thrombosis of origin of the celiac trunk. C Right: Pervious vessels of the celiac trunk. Case 1. D Left: Re‐canalized superior mesenteric artery. E Middle: Sub‐renal aortic thrombosis. F Right: Gangrenous gallbladder. Case 2. G Left fused duodenum and abdominal aorta at the level of aneurysm, note fluid and air accumulation within aneurism cavity and aorto‐bi‐iliac endoprosthesis. Case 3. H Middle: Edema of sigmoid colon with the perivisceral fluid collection. I Right: Thrombotic right hypogastric vein. [file JOIM-291-224-s002.tiff]

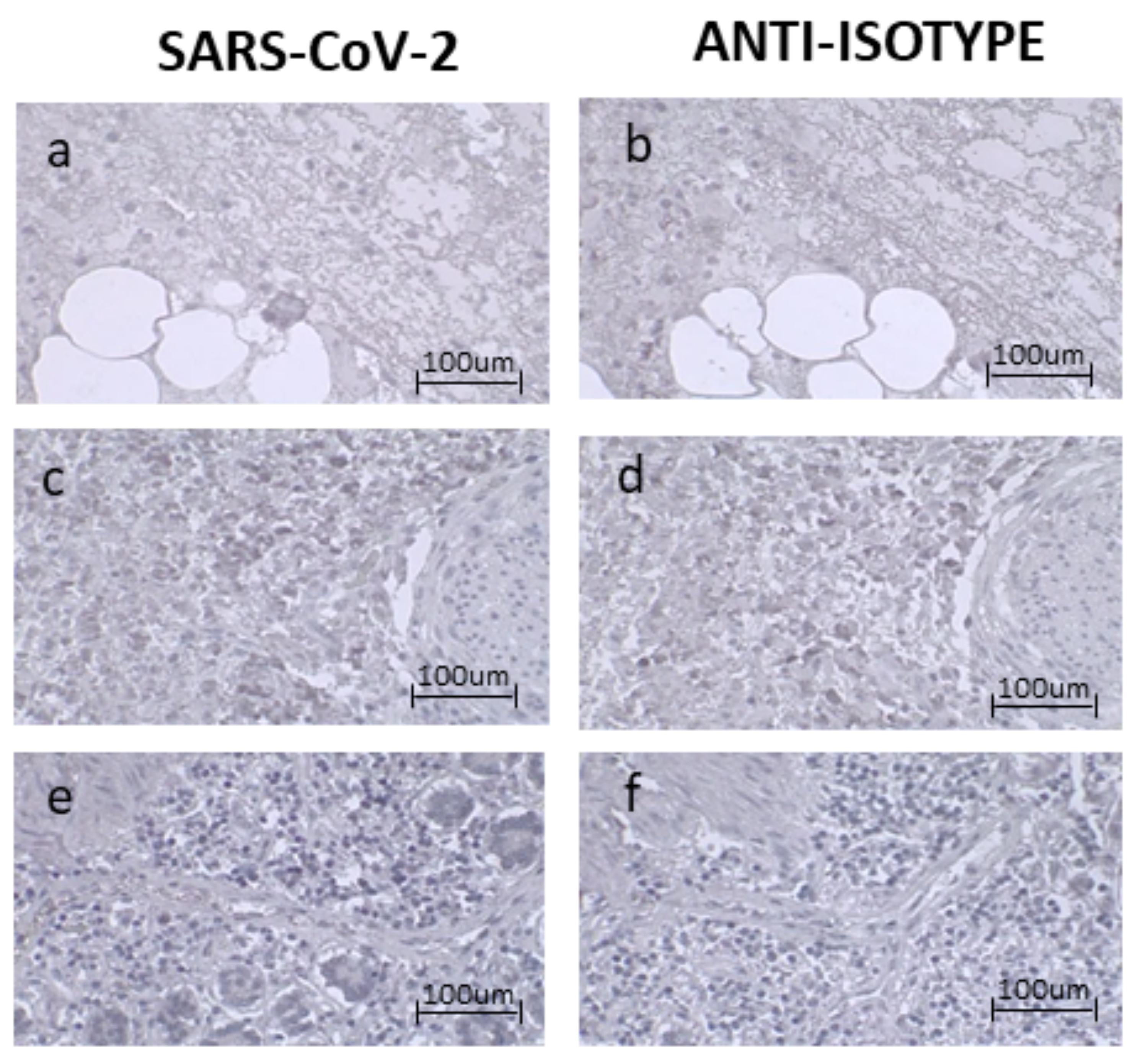

Supplement: Supplementary file 2 — Figure 2. Colon section. Optical microscopy. IHC 10X positive staining for (a) SARS‐CoV‐2 virus, (b) anti‐isotype staining. Cholecystitis section. Optical microscopy. IHC 10X positive staining for d) SARS‐CoV‐2 virus, e) anti‐isotype staining. Ileum section. Optical Microscopy. IHC 10X positive staining for g) SARS‐CoV‐2 virus, h) anti‐isotype staining. [file JOIM-291-224-s001.tiff]
